# Preview of 2013

**DOI:** 10.3402/ijch.v72i0.20474

**Published:** 2013-01-31

**Authors:** Kue Young

We would like to extend to our readers a Happy New Year for 2013. At this point, we can offer you a glimpse of what will appear in the *Journal* this year. We have planned at least two Special Issues. The first one is on Arctic Health in Russia, focusing on specifically two topics – occupational health and cancer epidemiology. Information on health in the Russian Arctic is limited, especially in the English language. This Special Issue will appear sometime in spring. Later in the year, a second Special Issue will be published on the proceedings of the 15th International Congress on Circumpolar Health, which was held in Fairbanks, Alaska, in August 2012. The Congress, held once every 3 years, presents a snapshot of what is currently engaging circumpolar health researchers, practitioners and policy makers.

The *Journal* would like potential authors to be aware of some types of articles that we are also interested in, besides Original Research Articles, Reviews and Short Communications. These are: History and Biography, Clinical Case Reports, Public Health Practice, Circumpolar Dissertations, and Conference and Workshop Reports. Brief descriptions of them can be found in the author guidelines on the website, but you are welcome to contact the Editor-in-Chief to discuss your proposed article to determine if it is within the scope of the journal.

*Kue Young* Editor-in-Chief

